# Analysis on the Moderating Effect of Innovation and Entrepreneurship Education Mode and Locus of Control of College Teachers and Students Based on Comic Style Recognition

**DOI:** 10.3389/fpsyg.2022.843665

**Published:** 2022-06-16

**Authors:** Bingjie Li, Kangshun Ren, Qiyang Guo, Xiaohong Huang, Jianjun Chen

**Affiliations:** ^1^School of Fine Arts, Nanjing Normal University, Nanjing, China; ^2^School of Design and Art, University of South China, Hengyang, China; ^3^School of Business, Gachon University, Seoul, South Korea; ^4^School of Information Management and Engineering, Shanghai University of Finance and Economics, Shanghai, China; ^5^Keyi College of Zhejiang Sci-Tech University, Hangzhou, China

**Keywords:** comics, innovation and entrepreneurship, education, locus of control, analysis

## Abstract

This study was carried out to explore the moderating effect of comic education and locus of control (LOC) in innovation and entrepreneurship education in colleges and universities. Firstly, the theoretical knowledge of comic education, innovation and entrepreneurship education, and LOC was briefly introduced, and the significance of comics for innovation and entrepreneurship education was discussed. Secondly, the existing innovation and entrepreneurship education modes in colleges and universities in China were introduced. Thirdly, a simple comparative analysis was conducted on the internal and external characteristics of LOC. Finally, an investigation was performed on the innovation and entrepreneurship ability of college students. The results demonstrate that the average score of students’ innovation spirit is 3.302, with a standard deviation of 0.481, suggesting that the current college students’ overall innovative spirit is moderate. Besides, students get moderate scores in each dimension, and the difference between different students is slight. Among them, the mean of reflectiveness is the highest (*M* = 3.446, *SD* = 0.540), and the mean of criticality is the lowest (*M* = 3.160, *SD* = 0.481). The average score of the current students’ entrepreneurial ability is 3.112, indicating that the students’ entrepreneurial ability is above the average. From the perspective of each dimension, students have the lowest score in opportunity discovery ability, which is at a low level (*M* = 2.821, *SD* = 0.873), while the other five dimensions are at a medium level. The highest is strategic decision-making ability (*M* = 3.264, *SD* = 0.749). At the same time, factors such as gender, grade, institution, and students’ relatives significantly impact students’ innovation and entrepreneurship ability. It can be concluded that colleges and universities should focus on controlling the quality of innovation and entrepreneurship teaching and guiding and carrying out innovation and entrepreneurship practice activities. They should also encourage students to participate in courses and activities related to innovation and entrepreneurship to improve college students’ innovation and entrepreneurship ability.

## Introduction

In order to cultivate students’ innovation consciousness and entrepreneurial ability and enable students to adapt to the social situation and meet the national economic needs, China has vigorously carried out the education of innovation and entrepreneurship. Unlike employment education, innovation and entrepreneurship education in colleges and universities aims to lay the foundation for innovation and entrepreneurship for college students, increase the proportion of college students’ entrepreneurship, and alleviate the employment pressure in China. Meanwhile, it can cultivate students’ innovative consciousness, help them accommodate society, and improve their working competence (Secundo et al., 2021). College students have substantial flexibility, and innovation and entrepreneurship education can guide their thinking and shape their spirit of innovation and entrepreneurship. Cultivating students’ creative and entrepreneurial ability is also an essential task of colleges and universities. It is a means to improve the visibility of colleges and universities, cultivate students, and improve the capability and literacy of students (Yan et al., 2019).

Miranda et al. (2020) believed that the progress of entrepreneurship education and research in colleges and universities had contributed to the growth of literature in related fields. Various perspectives have led to conflicts and challenges of multiple concepts. They put forward critical views on identified conflicts and challenges. In addition, based on the definition of entrepreneurship in education and the role of ethics in entrepreneurship teaching and evaluation, they identified seven questions to clarify the meaning of entrepreneurship in the context of engineering education. Handayati et al. (2020) explored the decisive role of entrepreneurship education on students’ entrepreneurial intention and examined the new role of entrepreneurial mentality between the two. They found that entrepreneurship education positively impacted students’ entrepreneurial choice and entrepreneurial mentality, and entrepreneurial mentality had successfully contributed to the relationship between entrepreneurship education and students’ entrepreneurial intention. Mittal and Raghuvaran (2021) stated that entrepreneurship education could cultivate learners’ ability, improve their ability to integrate theory with practice, and enhance their ability to develop in enterprises. Moreover, under the current educational background, the application of e-learning courses could facilitate the sustainable employment ability of college students, which was beneficial to the development of the national economy. Comics have cultural and hidden education characteristics; they are excellent educational carriers. With the rapid growth of new media in contemporary society, the Internet affects people’s work and lives even more profoundly. The form of comics is constantly changing, requiring educators to have enough sensitivity to capture the corresponding changes (Brott et al., 2010). The Chinese comics industry is still developing, and college students are more influenced by comics from Japan, the United States, and other countries. This requires educators to pay attention to domestic mainstream comics and master comics widely spread in China from Japan, the United States, and other countries. Educators should learn from its influence on contemporary college students to carry out feasible and effective educational activities.

This paper expounds on the basic theory of comic education, innovation and entrepreneurship education, and Locus of Control (LOC). Besides, the specific situation of China’s existing innovation and entrepreneurship education is researched to understand the influencing factors of its innovation ability and entrepreneurship ability to improve China’s innovation and entrepreneurship education system. First, the theoretical background of comic education, innovation and entrepreneurship education, and LOC is briefly introduced. Then, the significance of comics to innovation and entrepreneurship education is discussed. Secondly, Chinese colleges and universities’ existing innovation and entrepreneurship education models are introduced. Finally, a simple comparative analysis is conducted on the internal and external characteristics of the LOC to investigate the innovation and entrepreneurship ability of college students.

## Related Concepts and Methods

### Comic Education

The innovative practice of comic education has clear ideas and a solid foundation. It can bring into play the advantages of collaborative education combined with the characteristics of disciplines and majors. Comic education is both farsighted and practical. It has formed a demonstration and has good educational results, worthy of further promotion. The follow-up development should focus on the following two aspects of exploration and demonstration (Chen, 2019).

On the one hand, it is necessary to strengthen the theoretical depth and practical breadth of comic education. “The innovation of aesthetic education lies in reflecting on the problems existing in the traditional approach and emphasizing the selection and optimization of various approaches such as curriculum education, practical education, environmental education, and cultural education (Chen et al., 2020).” The aesthetic of creators of comics and their derived visual works can receive aesthetic education mainly manifested in the conception process, data collection, learning, thinking, etc. The viewers can get aesthetic education, primarily reflected in the resonance of the mind, the reflection of images in the brain, and the link of causing thinking (Feng and Chen, 2020; Liu et al., 2021c). By now, comic creators are generally art college students. It is necessary to carry out popular education and training in comic creation through public art education in universities, student associations, student self-organization, etc. to continuously cultivate non-art college students with comic appreciation hobbies and practical creation ability (Deng et al., 2021). In creating derivative works of comics, such as micro-film, animation production, etc., it is feasible to organize a multi-professional team to attract non-art college students to carry out creative practice (Zheng et al., 2018; Wu W. et al., 2020).

On the other hand, new online media can serve comic education. Xi Jinping pointed out, “The right way for new media ideological and public opinion work is to dissolve negative effects, stimulate positive energy, and become assistants in governing the country and building consensus (Zheng and Liu, 2020).” Mass media are increasingly relying on online channels. To grasp the initiative in new media, the comic education project shall use new media with high popularity and broad audiences, such as WeChat and microblog, and maximize the use of social media based on a smartphone as the communication channel, to achieve a fast, wide-ranging, and open effect. The project team is currently organizing and instructing students to independently develop and try out the comic education APPs to disseminate excellent and socialist education cartoons from a broader range (Liu et al., 2019; Liu and Chen, 2021).

**FIGURE 1:**
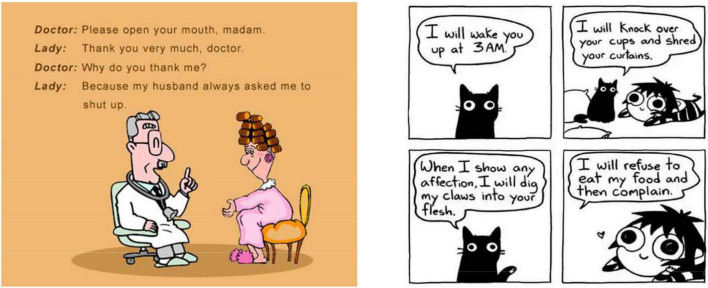
Caricature representation. Reproduced with permission from (Merriam-Webster), available at (https://www.merriam-webster.com/dictionary/caricature).

Comics and derivative visual works are full of visual impact, vivid, and intuitive. Images can make a deep impression on the human brain. The symbolic elements of comics also have a robust psychological hint on creators’ and viewers’ self-education and thinking depth (Liu et al., 2021a). Comics for educating people are a kind of meaningful and perceptible “tacit knowledge,” which is undoubtedly more in line with their cognitive characteristics and personality traits for contemporary college students (Ak et al., 2020). Figure 1 displays the expression form of cartoons.

### Innovation and Entrepreneurship Education

Innovation is the primary driving force for development. Entrepreneurship is a vital driving force for national economic growth and social value creation. Innovation and entrepreneurial activities play the role of bridges and links to transform science and technology into productivity (Liu et al., 2021b). The process of economic integration in the world is accelerating day by day. As the scientific and technological revolution and industrial transformation emerged, the international financial landscape also changed dramatically. Facing changes in the macro-environment, various countries have gradually realized that they should promote the manufacturing industry from factor and investment-driven to innovation-driven and use technological innovation to add new vitality to the manufacturing industry (Takemoto and Oe, 2021). Innovation has become a critical strategy for international competition and a driving force for the development of various countries. China is the world’s manufacturing superpower, yet China’s manufacturing industry is “comprehensive but not sophisticated, large but not strong” under such an era background. Besides, the Demographic Dividend is gradually disappearing, slowing down China’s economic development (Ting, 2019). The State Council put forward the “Made in China 2025” action program. The strategy emphasizes the need to place innovation at the core of the overall development of the manufacturing industry, rely on innovation to realize industrial transformation and upgrading, and realize the leap from “made in China” to “created in China.” The innovation-driven development strategy is critical to lead China’s economy toward high-speed and high-quality progress. It is necessary to fully stimulate the enormous potential of innovation in a wider range, more expansive space, and deeper fields and promote the construction of an innovative country and a world power in science and technology (Ting, 2019).

Talent is the main body of innovation. The driving force of innovation is essentially the driving force of talents. The key to talent training lies in colleges and universities. Talent is an essential element of innovation and development. As one of the critical factors to promote the rapid growth of a country’s economy and society, talent has a very prominent role and status in this era. The competition of economy, science, technology, and comprehensive national strength boils down to the competition of talents. Only when a country has multitudes of high-quality, innovative talents can it produce first-class innovation results and truly take the lead in innovation in international competition (Wang et al., 2019). At this stage, China is facing a leap-forward development, with opportunities and challenges coexisting, which has a far greater demand for innovative talents than other countries. As an important base for cultivating and transporting talents for society, colleges and universities naturally have to undertake this critical historical mission. Innovation and entrepreneurship education in colleges and universities is a necessary measure for China’s higher education to comply with the major strategy of developing an innovative economic system and building an innovative country (Heaney et al., 2019). As a frontier group accepting new ideas, concepts, and technologies, college students are the masters of the future society, the builders and promoters of national development, and the main participants and beneficiaries of innovation and entrepreneurship education in colleges and universities. Through entrepreneurship and innovation education, colleges and universities stimulate students’ innovative thinking, shape their innovative personality, encourage them to innovate and start businesses, help them achieve self-development, improve their comprehensive quality, and provide a solid intellectual support for constructing an innovative country (Castro et al., 2019). The Ministry of Education issued the *Opinions on Vigorously Promoting Innovation and Entrepreneurship Education in Colleges and Universities and Independent Entrepreneurship Work for College Students* in 2010. Since then, entrepreneurship and innovation education has received unprecedented attention and have rapidly developed. With the continuous improvement of entrepreneurship and innovation education, people are paying increasing attention to the effect of entrepreneurship and innovation education (Badri and Hachicha, 2019).

**FIGURE 2:**
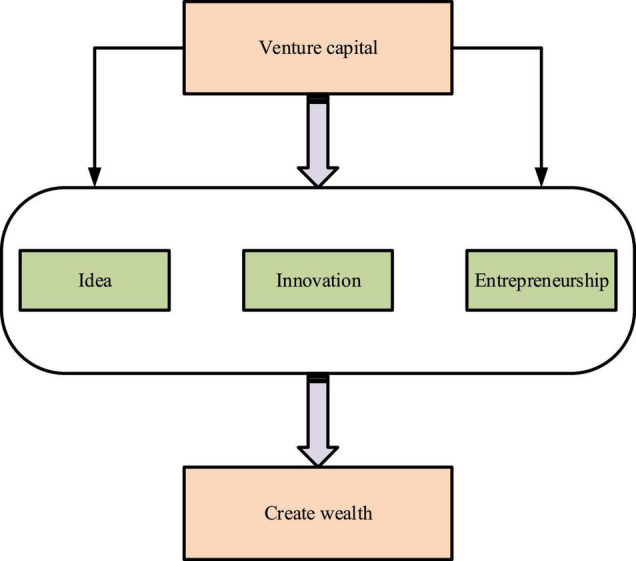
Elementary objects of innovation and entrepreneurship education in colleges and universities.

However, there is a lack of detailed research and substantial evidence on the effectiveness of entrepreneurship and innovation education. Entrepreneurship and innovation education has developed for over 20 years under the great attention and vigorous promotion of the Communist Party of China (CPC) and the national government. All parties need to study and reflect on the effectiveness and existing problems to promote the healthy development of entrepreneurship and innovation education. Of the 34,576 articles on the theme of “innovation and entrepreneurship education” on CNKI, only 167 researched the effectiveness of “innovation and entrepreneurship education” effectiveness. It can be seen that there is insufficient research on the effectiveness of entrepreneurship and innovation education. Figure 2 shows the primary objectives of innovation and entrepreneurship education according to the vision of the Innovation and Entrepreneurship Education Alliance of China.

Numerous studies have shown significant differences between men and women, especially in entrepreneurial motivation, industry choice, financing channels, etc. (Gerwin, 1988). Due to different social roles, men pay more attention to their rights, social status and honor, while women are more inclined to focus on family building. College students with different college levels and professional backgrounds also have significant differences in their thinking and behavior patterns. In addition, there are also differences among students of different grades. First-year students usually lack precise career planning at the beginning of college life. In contrast, senior students gradually have a clear plan due to their adaptation to college life and timely guidance from teachers. These factors also influence the behavior of college students (Heilman et al., 2003). Therefore, this paper proposes the first hypothesis.

H1: differences in gender, grades, institutions, and relatives will make college students’ entrepreneurial motivations significantly different.

### Locus of Control

The economic environment is becoming even more turbulent under the macro background of economic globalization and China’s new normal economic development. In addition, China’s economic transformation has encountered bottlenecks. Many innovations have not been transformed into actual productivity, and labor-intensive industries still occupy the central position of the economy. Moreover, China has a large working population globally, but social work posts are limited. With the implementation of expanding enrollment in public high schools, the number of graduates has repeatedly hit new highs. Consequently, problems such as employment difficulties and structural unemployment have become increasingly severe (Wu and Wu, 2017; Zheng and Ke, 2020).

As early as 1985, the management scientist Drucker proposed that when the economy was under pressure, the transformation of the economic system from a “managerial economy” to an “entrepreneurial ingenuity” could enable the economy to cross the rapids. Today, the economic and social benefits of entrepreneurship have been extensively recognized worldwide. The practice has confirmed that entrepreneurial activities are conducive to improving social productivity, providing abundant employment opportunities for the society, and promoting the transformation of new technologies and new ideas into actual productivity; it is crucial to the long-term sustainable development of the economy (Wu and Song, 2019). College students’ participation in entrepreneurship can avoid the waste of educational resources and drive the progress of high-tech industries, thereby accelerating the transformation of China’s economic model from labor-intensive to knowledge-intensive and promoting healthy economic development.

The 16th, 17th, and 18th National Congress of CPC have emphasized the importance of entrepreneurial activities and have raised “encouraging job growth through the creation of new businesses” to the strategic height of the CPC and the country (Wu Y. J. et al., 2020). In August 2015, the Ministry of Education, the Ministry of Human Resources and Social Security, and the State-owned Assets Supervision and Administration Commission jointly awarded the first 50 Practice Education, Innovation and Entrepreneurship Base of National Universities. This batch included 33 universities directly under the Ministry of Education, seven local undergraduate colleges, and two high vocational colleges. It can be seen that Chinese college students have unique entrepreneurial advantages (Zheng et al., 2015; Yuan and Wu, 2020).

However, as Reuters, one of the world’s four major news agencies, reported in October 2015, “only a minimal number of Chinese college students can succeed in entrepreneurship.” Throughout the truth, the success rate of college students’ entrepreneurship is only about 1%. The problem of original motivation is still the root cause: the uncertainty and infirmity of college students’ entrepreneurial motivation.

Entrepreneurial motivation is the premise and foundation of entrepreneurial behavior and is essential for entrepreneurial research. Existing research on entrepreneurial motivation is primarily aimed at entrepreneurs, a new generation of migrant workers, etc. The content structure of entrepreneurial motivation of college students is significantly different from that of other groups, which is worthy of further research. In addition, there is no mature measurement tool for college students’ entrepreneurial motivation in the existing literature. Most research on the influencing factors of college students’ entrepreneurial motivation is qualitative. It lacks empirical tests, which is not conducive to selecting, evaluating, and training college students’ entrepreneurship.

Entrepreneurial motivation is the product of individual and environmental factors and is affected by differences in individual characteristics, such as self-efficacy and LOC. However, few studies focus on the influence of entrepreneurial self-efficacy and LOC on entrepreneurial motivation. As an essential personal factor, LOC also plays a vital role in entrepreneurial motivation.

**FIGURE 3:**
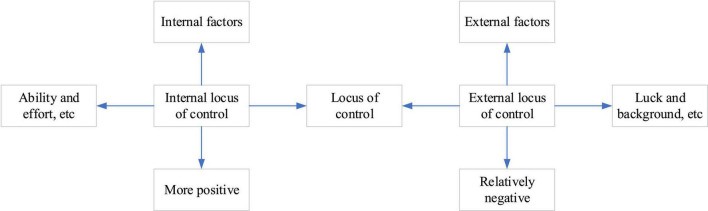
Locus of control.

Locus of control determines the perpetrator’s view of the consequences of his behavior, such as internal LOC. The perpetrator’s ability and effort determine the consequences of the perpetrator. As the saying goes, man can conquer nature; just look for the corresponding path for success, not for failure. This proverb is a high generalization of internal LOC. On the contrary, the external LOC believes that the result of behavior is determined by factors such as luck and background, and these factors overshadow all efforts and struggles. Figure 3 reveals the difference between the internal and external LOC.

Entrepreneurial self-efficacy is an individual’s cognitive evaluation of one’s ability and an entrepreneur’s belief and self-confidence in accomplishing predetermined entrepreneurial goals, which can predict entrepreneurial activities. Even if potential entrepreneurs have similar skills and traits, they have various entrepreneurial motivations, entrepreneurial behavior decisions, entrepreneurial field choices, and different degrees of entrepreneurial tendencies. These phenomena are all related to the differences in potential entrepreneurs’ entrepreneurial self-efficacy. Individuals with high levels of entrepreneurial self-efficacy also have relatively high positive evaluations of themselves. Therefore, they have high entrepreneurial expectations, a solid ability to predict risk, and strong confidence in completing entrepreneurial activities. Accordingly, this paper proposes the second hypothesis.

H2: college students’ LOC is regulatory in stimulating entrepreneurial self-efficacy to entrepreneurial motivation.

### Survey Design

This questionnaire is mainly composed of four parts. The first part is the basic information of the respondents, including gender, grade, school type, primary type, and family location, testing whether the observed variables are significantly different in different populations. The second part is the degree of participation and feelings of students in various forms of entrepreneurship and innovation education. The third part is the *College Students’ Innovative Spirit Scale* to measure the innovative spirit of the respondents. The fourth part is the *College Students’ Entrepreneurial Ability Scale* to observe the students’ entrepreneurial ability.

#### College Students’ Innovative Spirit Scale

The *College Students’ Innovative Spirit Scale* compiled by Wang Hongli and Liu Hong in 2009 is adopted to understand the state of students’ innovative spirit. The questionnaire includes four dimensions: flexibility, novelty, criticism, and reflection, with a total of 25 questions. Among them, questions 1, 4, 6, 12, 15, 16, and 22 constitute the dimension of flexibility, questions 2, 8, 17, 19, 22, 23, and 25 form the dimension of unconventionality, and questions 3, 7, 11, 13, 18, and 20 constitute the critical dimension, while questions 5, 9, 10, 14, and 24 form the reflective dimension.

Questions 4, 11, 13, 14, 20, 22, and 25 are scored in reverse to prevent the research subjects from having a mindset affecting the measurement results. The scale adopts a five-point Likert scoring method. The options are “completely inconsistent,” “not quite consistent,” “not sure,” “relatively consistent,” and “completely consistent” assigned 1 ∼ 5 points respectively. This paper uses the average level of the total score of the scale to measure the level of innovation spirit. Specifically, 1–2 points are low level, 2–3 points are middle and lower level, 3–4 points are middle and upper level, and 4–5 points are high level.

#### College Students’ Entrepreneurial Ability Scale

The *College Students’ Entrepreneurial Ability Scale* was compiled by Yang and Department (2017). There are 22 questions in total from six aspects: opportunity exploration ability, organizational management ability, strategic decision-making ability, resource integration ability, innovation and creation ability, and frustration endurance ability. Among them, questions 1, 2, 3, and 4 constitute the dimension of opportunity exploration capability; questions 5, 6, and 7 form the dimension of organizational management ability; questions 8, 9, and 10 constitute the dimension of strategic decision-making ability; questions 11, 12, 13, and 14 include the dimension of resource integration ability; questions 15, 16, 17, and 18 constitute the dimension of innovation and creation ability; questions 19, 20, 21, and 22 form the dimension of frustration endurance ability. The scale uses a five-point Likert scoring method, with “completely inconsistent,” “not quite consistent,” “not sure,” “relatively consistent,” and “completely consistent” assigned 1–5 points, respectively. The average level of the total score of the scale is used to measure entrepreneurial ability. 1–2 points are low level, 2–3 points are middle and lower class, 3–4 points are middle and upper class, and 4–5 points are high level.

#### Original Scale

This survey classifies entrepreneurship and innovation education into three categories concerning relevant literature: theoretical courses of entrepreneurship and innovation, practical activities of entrepreneurship and innovation, and professional courses integrated into entrepreneurship and innovation education. The original scale sets up 18 questions to investigate students’ evaluation and participation in three different forms of entrepreneurship and innovation education.

### Reliability and Validity of Scales

#### Reliability

Reliability is an indicator of the reliability of the measurement results and is generally tested by Cronbach’s α coefficient. The Cronbach’s α coefficient value usually ranges from 0 to 1, with higher values indicating firmer internal consistency across a range of items. Generally, Cronbach’s α coefficient above 0.6 is considered high reliability.

#### Validity

Validity refers to how a measurement tool or method can accurately measure what it needs to measure. The questionnaire used here is compiled through discussions and repeated revisions by experts and teachers from various parties, ensuring that the questionnaire has good content validity. Bartlett’s Test of Sphericity is used to re-examine the construct validity of the questionnaire to confirm the validity.

## Research Results and Analysis of Entrepreneurship Education Based on Comic Style and Locus of Control

### Specific Investigation

**FIGURE 4:**
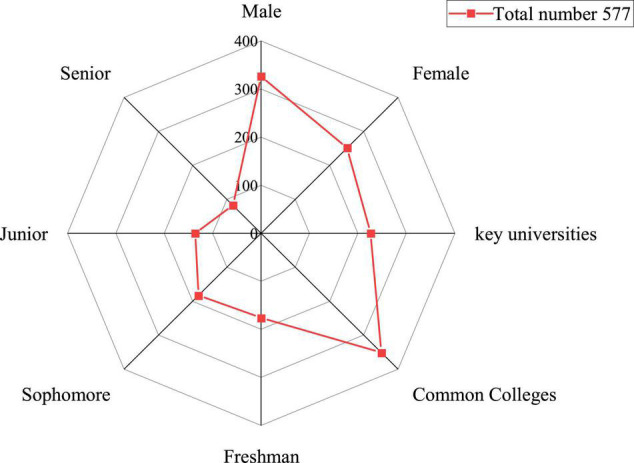
Composition of investigated students.

A questionnaire survey is conducted on college students in some colleges and universities based on the above theoretical knowledge. Due to the impact of COVID-19, the survey is carried out online. The basic information and students’ attitudes to innovation and entrepreneurship education are investigated from multiple aspects, and statistical analysis is implemented according to the survey results. A total of 700 questionnaires are distributed, and 603 questionnaires are finally recovered. After investigation, there remain 577 valid questionnaires, and the effective recovery rate of the questionnaire is 82%. Nearly 70% of the students have served as student leaders, of which only 16.2% have entrepreneurial experience. Most of the students have no entrepreneurial experience. In short, more than half of the students have no entrepreneurial intention. Besides, some students have entrepreneurial plans in 2–3 years and more than 5 years. Figure 4 shows the sample structure.

The questionnaire adopts the average score system. In other words, the answers to the questionnaire questions correspond to 1–5 points, with the lowest score of 1 and the highest score of 5. The average value of all students in the survey project is calculated to obtain the standard of the overall college students for the project, and the corresponding standard deviation is estimated to get the specific level of innovation and entrepreneurial ability of college students.

### Results of Reliability and Validity

#### Reliability Analysis

**FIGURE 5:**
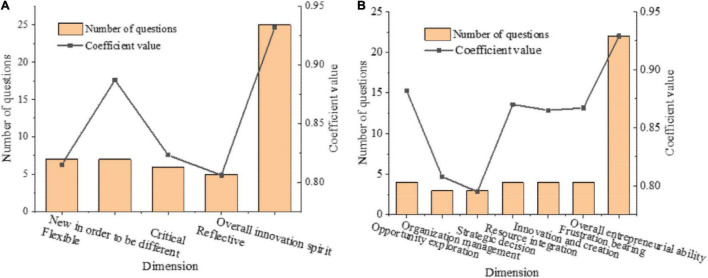
Reliability test results (**A:** reliability test of the *College Students’ Entrepreneurial Spirit Scale*; **B:** reliability test of the *College Students’ Entrepreneurial Ability Scale*).

Figure 5 provides the reliability test results of the *College Students’ Innovative Spirit Scale* and the *College Students’ Entrepreneurial Ability Scale*.

In Figure 5, the Cronbach’s α coefficient of the overall and each dimension of the innovative spirit is above 0.6, indicating a high degree of internal consistency and good reliability.

The Cronbach’s α coefficient of the overall and each dimension of the entrepreneurial ability is above 0.6, indicating a high degree of internal consistency and good reliability.

#### Validity Analysis Results

Table 1 lists the results of the Kaiser–Meyer–Olkin (KMO) Measure and Bartlett’s Test of Sphericity of two scales.

**TABLE 1:** Validity analysis results.

		Innovation spirit	Innovation ability
KMO Measure of Sampling Adequacy		0.924	0.939
Bartlett’s Test of Sphericity	Approximate chi-square	4371.114	3808.288
	Degree of Freedom	300	66
	Significance	0.000	0.000

In Table 1, the KMO results of each scale are more significant than 0.8, and the Bartlett results are 0.000 (<0.001), showing good adaptability. This result proves that the questionnaire has excellent construct validity in satisfaction.

### Analysis of the Research Results of Innovation Capability and Entrepreneurial Competence

**FIGURE 6:**
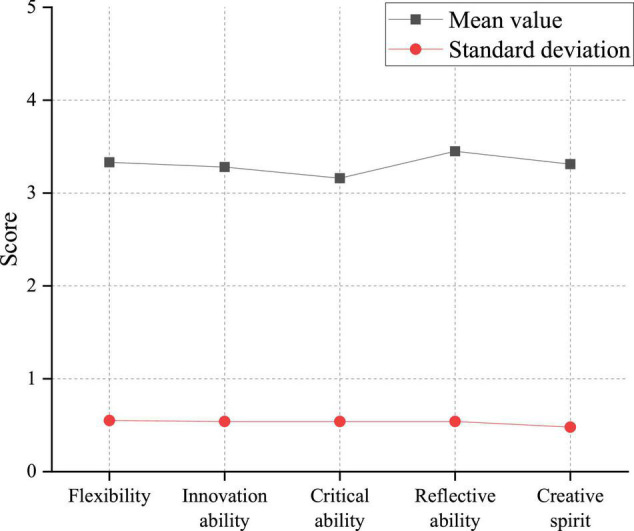
Analysis of college students’ innovation capability.

The survey of students’ innovation and entrepreneurship ability separately analyzes college students’ innovation capability and entrepreneurial competence. The investigation of students’ innovation capability is divided into four dimensions: flexibility, innovation ability, critical ability, and reflective ability. Figure 6 displays the statistical results.

Figure 6 suggests that the average score of students’ innovative spirit is 3.302, and the standard deviation (SD) is 0.481. From the mean value (M), the current college students’ overall innovation spirit is medium. From the perspective of each dimension, the four dimensions are all in the middle level, and the differences in different students are small. Among them, the mean of reflectiveness is the highest (*M* = 3.446, *SD* = 0.540), and the mean of criticality is the lowest (*M* = 3.160, *SD* = 0.481).

**FIGURE 7:**
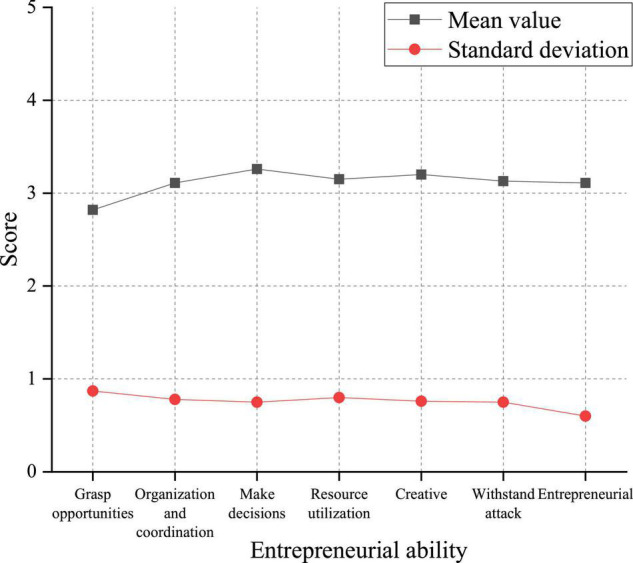
Analysis of entrepreneurial competence of college students.

The overall students’ innovation capability is at a medium level, and their reflective ability is strong, indicating that students can independently think and plan innovative activities in the long run. However, the low score of critical ability in Figure 6 demonstrates that college students have insufficient capacity to break the limitations of the traditional mindset. This will also make students lose their innovative consciousness in the face of the seemingly impossible mission, which requires attention. Similarly, the entrepreneurial competence of college students is researched from six dimensions: the ability to grasp opportunities, the organization and coordination ability, the ability to make decisions, the resource utilization capability, the creative ability, and the ability to withstand blows. The survey results are presented in Figure 7.

In Figure 7, the average score of the current students’ entrepreneurial ability is 3.112, indicating that the students’ entrepreneurial ability is at an upper-middle level. From the perspective of each dimension, students have the lowest score in opportunity discovery ability (*M* = 2.821, *SD* = 0.873). The other five dimensions are in the middle level. Students has the highest level in the strategic decision-making ability (*M* = 3.264, *SD* = 0.749).

Figure 7 signifies that the score of students’ entrepreneurial competence is slightly lower than that of innovation capability, but it is still at a medium level. It is worth noting that the students’ ability to grasp opportunities is low, indicating that they lack specific life experience and are not sensitive to prospects, which will be improved with the expansion of their expertise. The students get the highest score of the ability to make decisions, proving that students generally have strong self-confidence to make decisions more decisively.

### Analysis of the Influence of External Factors on Innovation and Entrepreneurship Ability

**FIGURE 8:**
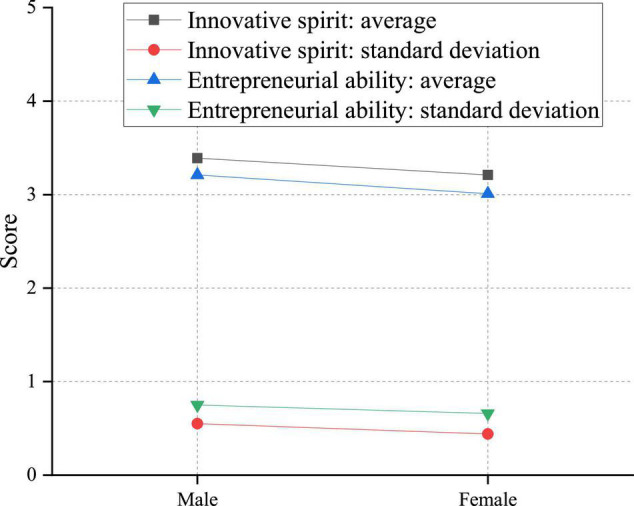
An analysis of the impact of gender difference on innovation and entrepreneurship ability of college students.

There are many influencing factors of students’ innovation and entrepreneurship ability. Some primary influencing factors are taken into consideration by this investigation. Firstly, at the moment of serious gender confrontation, the gender difference in innovation and entrepreneurship ability of college students is investigated. Figure 8 provides the specific research results.

According to Figure 8, there are significant gender differences in the innovative spirit of college students. The specific performance is that the creative energy of males is higher than that of females. There may be two reasons for this result. First, males are generally lively and active, while females are usually quiet. Especially in adolescence, males dare to innovate and break the rules. In contrast, females are primarily obedient and abide by the rules, which is not conducive to improving their innovative spirit. Second, the increasing rise of women’s status has pressured males. Therefore, males need to break through the limit and innovate to seize the opportunity and improve their competitiveness, which leads to males’ innovative spirit being higher than females.

There are also significant gender differences in the entrepreneurial ability of college students. The entrepreneurial power of males is higher than that of females. The main factor may be the personality difference between different genders. Males usually have more robust innovation and entrepreneurial intention, risk tolerance, and self-achievement motivation than females. Therefore, they can actively participate in related activities and improve their entrepreneurial ability. In terms of employment and career selection, girls prefer stable and low-risk jobs, so they will not deliberately improve their entrepreneurial ability. In most cases, males have a more adventurous spirit when thinking about problems, while females mostly pay more attention to stability. Therefore, their adventurous spirit is significantly lower than males of the same age.

**FIGURE 9:**
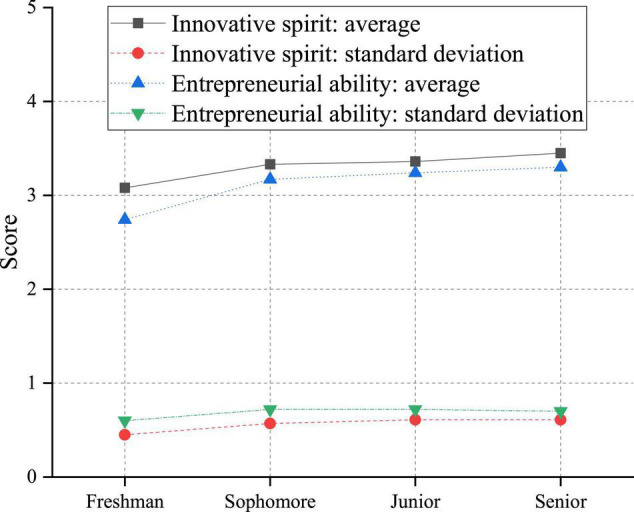
Analysis of the impact of grade difference on innovation and entrepreneurship ability of college students.

According to Figure 8, male students are significantly better than female students from the perspective of innovation spirit and entrepreneurial ability, which shows that the gender difference has a considerable impact on college students’ innovation and entrepreneurial ability. In most cases, females are more adventurous in thinking, while most males pay more attention to stability, and they are less adventurous than females of the same age. In addition to gender differences, a comparative analysis is also performed on the age difference, which is reflected by the grade instead of the specific period of students. Figure 9 describes the detailed survey results.

**FIGURE 10:**
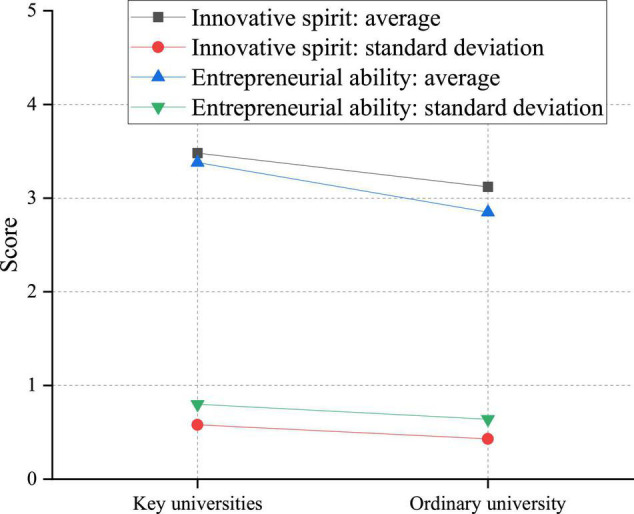
An analysis of the impact of college differences on the innovation and entrepreneurship ability.

According to Figure 9, both innovation capability and entrepreneurial competence show an increasing trend with the grade increases. The innovation ability of high-grade students is significantly higher than that of low-grade students. It can be seen that low-grade college students have lost some innovation and entrepreneurship ability under the cramming examination-oriented education. With the advancement of university courses, their innovation and entrepreneurship abilities are gradually restored. Up to the high grade, the score of innovation and entrepreneurship ability is about 0.5 more elevated than the average of low-grade students, which shows the influence. After comparing grade differences, the effects of different colleges and universities are analyzed, and the results are shown in Figure 10.

**FIGURE 11:**
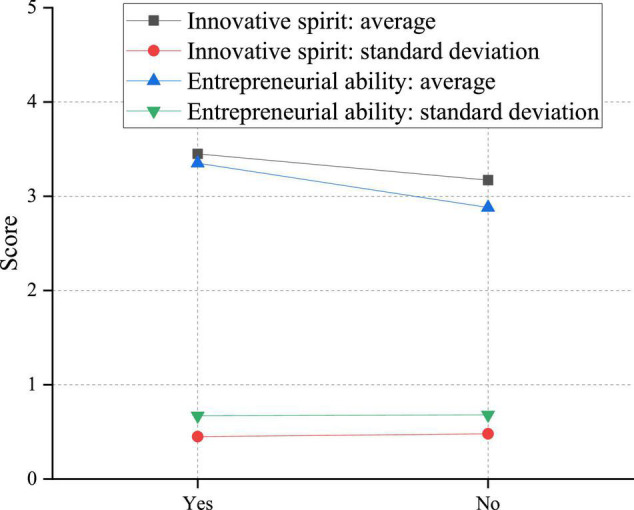
Analysis of the impact of family differences on innovation and entrepreneurship ability of college students.

Figure 10 shows that the innovation capability and entrepreneurial competence of students in 985 engineering schools are significantly better than those of students in ordinary schools, indicating that students’ ability and learning environment have a critical impact on their innovation and entrepreneurship ability. Outstanding personal skills can motivate vital innovation, creativity, and possibilities in a good learning environment. It is not difficult to explain the tremendous amount of money that the state allocates to key institutions each year. In addition, the impact of whether relatives of college students have started a business on their innovation and entrepreneurship ability is discussed, and the results are shown in Figure 11.

Evidently, students with relatives starting their businesses have more vital innovation and entrepreneurship abilities. Under the influence of relatives, these students will have inherent ideological advantages. They have early ideological preparation that other students do not understand the knowledge of innovation and entrepreneurship deeply and unconsciously. In contrast, students without relatives starting a business may think that entrepreneurship has nothing to do with them and will not take the initiative to accept the knowledge related to innovation and entrepreneurship.

### Analysis of the Impact of Students’ Individual Factors on Innovation and Entrepreneurship Ability

**FIGURE 12:**
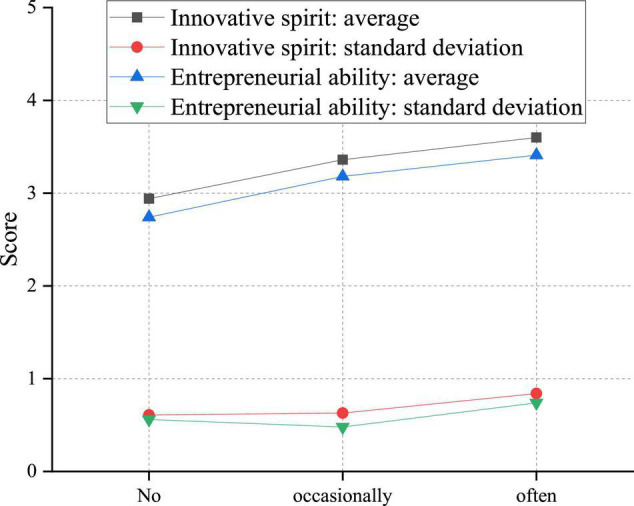
Influence of college students’ participation in theory courses on innovation and entrepreneurship ability.

In addition to the above uncontrollable factors, the influence of relatively controllable individual factors on students’ innovative and entrepreneurial ability should also be considered. Here, the survey is performed on the influence of college students’ participation in related theoretical courses and practical activities of innovation and entrepreneurship on students’ innovation and entrepreneurship ability. Figure 12 indicates the impact of academic classes on students’ innovation and entrepreneurship abilities.

**FIGURE 13:**
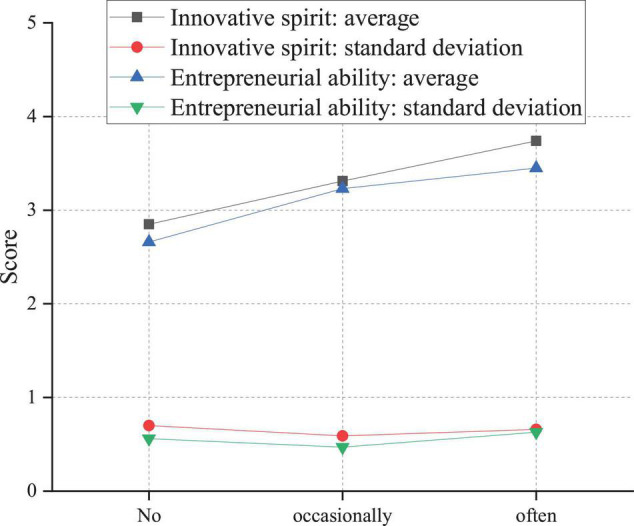
Influence of college students’ participation in practical activities on innovation and entrepreneurship ability.

There are compulsory and elective courses of innovation and entrepreneurship for college students, and different students show different situations of participating in the class. Therefore, it is discussed separately. From Figure 12, students who often join in the innovation and entrepreneurship theoretical course obtain the highest score, followed by students who occasionally participate in the innovation and entrepreneurship theoretical course. Those who do not participate in the innovation and entrepreneurship theoretical course get the lowest score. Therefore, the academic course of innovation and entrepreneurship is significant for cultivating college students’ innovation and entrepreneurship ability. Figure 13 illustrates the influence of practical activities on innovation and entrepreneurship ability.

Practical activities include entrepreneurship projects, entrepreneurship training, and entrepreneurship plan competition and always adhere to the principle of students’ voluntary participation. Therefore, there are some differences in the degree of the involvement of different students in practical activities. Thus, the one-way analysis of variance is conducted on the variables of innovative spirit and entrepreneurial ability of students with varying degrees of participation in practical exercises. In Figure 13, the average values of creatives’ innovative spirit who never/occasionally/often participate in innovation activities are 2.8531, 3.3088, and 3.7411, respectively, and the average values of entrepreneurial ability are 2.6621, 3.2272, and 3.4467, respectively, showing a gradually increasing trend. The *F* value of innovative spirit is 28.783, *P* < 0.01; the *F* value of entrepreneurial ability is 21.218, and *P* < 0.01, indicating significant differences. After comparison, students’ innovative spirit and entrepreneurial ability are related to the frequency of participating in courses and activities related to entrepreneurship and entrepreneurship education. Specifically, students who often participate in entrepreneurship and entrepreneurship education have the best innovative spirit and entrepreneurial ability, followed by students who occasionally participate. Those who do not participate rank the lowest.

### Influence of Students’ Perception of Innovation and Entrepreneurship Education in Colleges and Universities on Their Innovation and Entrepreneurship Ability

**FIGURE 14:**
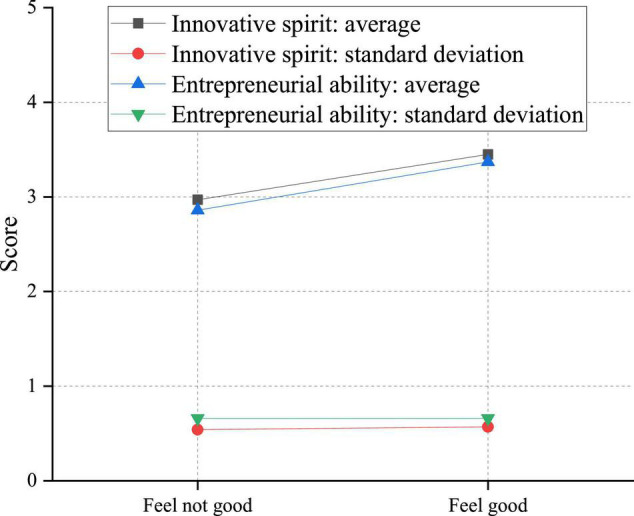
Influence of college students’ perception of innovation and entrepreneurship theoretical courses on their innovation and entrepreneurship ability.

Different colleges and different teachers will lead to various teaching effects. Consequently, different students will have different feelings about innovation and entrepreneurship education. Some students feel that they have gained a lot, but some have no gain. Therefore, students’ perception of innovation and entrepreneurship education is investigated to obtain the influence of different teaching qualities on students’ innovation and entrepreneurship ability. Firstly, the feelings of students who have experienced innovation and entrepreneurship theoretical courses and their innovation and entrepreneurship ability are analyzed. Figure 14 shows the detailed results.

It can be seen from Figure 14 that the mean value of the innovative spirit of students with a low sense of harvest is 2.9684; the mean value of entrepreneurial ability is 2.8617; the mean value of the innovative spirit of students with a high sense of harvest is 3.4495; the mean value of entrepreneurial knowledge is 3.3728; *P*-value is less than 0.01. It shows that improving students’ innovative spirit and entrepreneurial ability is related to the degree of harvest in the theoretical curriculum. Specifically, the students with a higher sense of harvest in the academic curriculum have a higher innovative spirit and entrepreneurial ability than the students with a lower sense of harvest.

Figure 14 demonstrates that the more satisfied the students are with innovation and entrepreneurship practical activities, the stronger their innovation and entrepreneurship ability is, which also explains the influence of self-satisfaction on their power. Specifically, the more students with strong innovation and entrepreneurship ability, the more they can play a corresponding role in innovation and entrepreneurship practical activities, which will increase the degree of recognition of students themselves. This effect is equivalent to the position of internal LOC so that they can play a better innovation and entrepreneurship strength and form a delicate cycle. Nevertheless, students with poor satisfaction with innovation and entrepreneurship practical activities will lead to effects similar to that of external LOC, resulting in negative emotions. These emotions can reduce their self-confidence to some extent, thereby reducing their innovation and entrepreneurship ability, resulting in low scores. In addition, the impact of the integration of innovation and entrepreneurship courses with other courses on the innovation and entrepreneurship ability of college students is analyzed.

**FIGURE 15:**
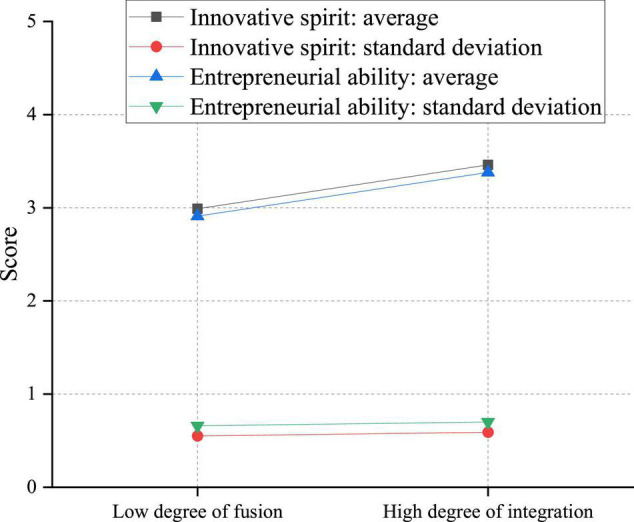
Impact of the integration of innovation and entrepreneurship courses with other courses on the innovation and entrepreneurship ability of college students.

Through Figure 15, students who believe that innovation and entrepreneurship education is highly integrated with other courses have stronger innovation and entrepreneurship ability. These students have acquired considerable knowledge of innovation and entrepreneurship in different classes with a more profound impression. The knowledge imperceptibly strengthens students’ innovation and entrepreneurship ability, reflected in the high score in Figure 15. However, those students who feel that the integration degree of the two is low do not obtain or only obtain very little knowledge related to innovation and entrepreneurship. Although the courses still have a recessive educational effect on them, the result is not prominent enough to increase the score.

### Analysis of the Moderating Effect of Locus of Control

The path analysis method of the structural equation model is used to construct the causal model path analysis of the regression analysis of various factors of college students’ entrepreneurial motivation on entrepreneurial self-efficacy, LOC, and the interaction between entrepreneurial self-efficacy and LOC. The two variables of entrepreneurial self-efficacy and LOC are centralized based on the total mean. The model constructed using Mplus software analysis and robust maximum likelihood estimation method can effectively estimate the residual estimator of dependent variables, making the constructed model more reasonable and the estimated coefficient strong. The standardized path coefficients and their determination coefficients in the model are summarized in Table 2.

**TABLE 2:** Regression coefficients of college students’ entrepreneurial motivation factors on entrepreneurial self-efficacy, LOC, and interaction terms.

Independent variable	Dependent variable	Beta	Standard error (*SE*)	*R* ^2^
Entrepreneurial efficacy	Independent motivation	0.644**	0.129	0.102
LOC		0.464**	0.177	
Interaction term		−0.621**	0.236	
Entrepreneurial efficacy	Self-actualization motivation	0.306*	0.128	0.138
LOC		–0.209	0.173	
Interaction term		0.127	0.232	
Entrepreneurial efficacy	Responsibility motive	0.641**	0.125	0.167
LOC		0.473**	0.17	
Interaction term		−0.501*	0.227	
Entrepreneurial effectiveness	Survival motivation	0.141	0.132	0.086
LOC		−0.384*	0.178	
Interaction term		0.147	0.238	
Entrepreneurial efficacy	Responding to policy motivations	0.229	0.13	0.106
LOC		0.183	0.176	
Interaction term		0.008	0.236	
Entrepreneurial efficacy	Seizing the opportunity motivation	0.755	0.127	0.133
LOC		0.744	0.173	
Interaction term		–0.837	0.231	

*Stands for 5% significance level and **stands for 1% significance level.

The results in Table 2 indicate that LOC plays a regulatory role in the impact of college students’ entrepreneurial self-efficacy on the independent motivation factor of entrepreneurial motivation of college students (regression coefficient β = –0.621, *SE* = 0.236, *P* < 0.01). Besides, LOC plays a moderating role in the influence of college students’ entrepreneurial self-efficacy on the responsibility motivation factor of entrepreneurial motivation of college students (regression coefficient β = –0.501, *SE* = 0.227, *P* < 0.05). In addition, LOC plays a moderating role in the influence of college students’ entrepreneurial self-efficacy on the factor of seizing the opportunity motivation (regression coefficient β = –0.501, *SE* = 0.227, *P* < 0.05). Moreover, the moderating effect of LOC on the self-realization motivation, survival motivation, and responding to policy motivation of college students’ entrepreneurial motivation factors is not significant. In other words, there is no moderating effect on these three factors.

The specific verification results of hypotheses are summarized in Table 3.

**TABLE 3:** Test results of hypotheses.

Number	Content	Result
H1	Differences in gender, grades, institutions, and relatives will make college students’ entrepreneurial motivations significantly different.	Tenable
H2	College students’ LOC plays a regulatory role in stimulating entrepreneurial self-efficacy to entrepreneurial motivation.	Tenable

## Discussion and Analysis

### Students’ Innovative Spirit and Entrepreneurial Ability

The average value of college students’ innovative spirit is 3.30, at a medium level. There is still a big gap from the high level. There may be the following reasons for this result.

First, school education plays a leading role in the physical and mental development of students. Before entering university, especially in high school, students receive exam-oriented education aiming at the scores. Exam-oriented education is concentrated on teachers’ textbooks, with a single teaching form. It usually adopts the teaching method and pays attention to learning knowledge, ignoring students’ initiative and potential, which is not conducive to cultivating students’ autonomous learning ability. The standardized examination and evaluation system has restrained the development of students’ personalities and creativity. In addition, school teachers prefer children who have good academic performance and don’t make trouble. These factors in schools have obliterated students’ imagination to a certain extent, making students lack the consciousness and courage of innovation.

Second, this has a particular relationship with the students’ living environment and peer pressure in relatively similar closed peer groups for an extended period. In adolescence, students’ outlook on life and world outlook is not mature, easy to shake, and produce conformity psychology. In the context of peer pressure and social group interaction, students are usually easier to give up some ideas, ideas, or behaviors they adhere to and make behaviors consistent with most people to integrate into this environment. Therefore, they often lack the courage to innovate.

Third, this also reflects that the educational synergy for cultivating students’ innovative spirit has not yet been formed. Developing students’ creative energy requires long-term efforts from schools to create an innovative campus atmosphere, parents and teachers to protect students’ curiosity, and society to accommodate the diversity of personalities. At present, students’ creative spirit is at the general level, just showing that all parties still need to continue to promote the formation of a joint educational force to develop innovative spirit.

The average value of college students’ entrepreneurial ability is 3.10, at a medium level. There is still a big gap from the high level. There may be the following reasons for this result.

First, entrepreneurial ability refers to a relatively complex comprehensive practical ability rather than registering a company or starting a business. It requires intelligence, a wealth of knowledge and experience, and the ability to grasp and identify opportunities, organize resources, take risks, and solve problems creatively. Colleges and universities are like ivory towers, disconnected from real society. Students live in a stable school environment, face fewer complex problems, and have fewer opportunities to practice. It is difficult for students to improve their ability to solve complex problems in practice, leading to a low level of entrepreneurial ability.

Second, it has a particular relationship with students’ entrepreneurial cognition and participation in innovative and entrepreneurial activities. When students have entrepreneurial intentions and ideas, they will actively participate in innovation and entrepreneurship-related activities to continuously improve their entrepreneurial ability in practice. On the contrary, if students do not have entrepreneurial ideas, they will not actively participate in relevant activities and naturally cannot improve their practical ability. This investigation found that students’ participation in innovation and entrepreneurship-related courses and activities is at a general level, making students’ entrepreneurial ability tend to be mediocre.

Finally, according to the teachable entrepreneurship theory, students’ entrepreneurial ability can be effectively improved through innovation and entrepreneurship education. The current low level of students’ entrepreneurial ability also reflects that the current school’s innovation and entrepreneurship education are not perfect, and there are specific problems needing further improvement.

### Differences in Gender, Grade, and Close Relatives’ Entrepreneurship Affect Students’ Innovative Spirit and Entrepreneurial Ability

There are significant gender differences in the innovative spirit of college students. The specific manifestation is that the innovative spirit of males is higher than that of females. On the one hand, males are generally livelier and more active, and females are quieter. In particular, adolescent males dare to be unconventional and break the rules. In contrast, females are primarily obedient and obedient and abide by the authorities, which is not conducive to improving their innovative spirit. On the other hand, the rising status of women brings certain pressure to males, so males need to break through the limits and innovate to seize the opportunity to improve their competitiveness. As a result, males have a higher innovative spirit than females.

There are also significant gender differences in the entrepreneurial ability of college students. Specifically, the entrepreneurial capacity of males is higher than that of females. The main factor may be the personality difference between different genders. Males usually have more substantial innovation, entrepreneurial intention, risk tolerance, and self-achievement motivation to participate in related activities actively and improve their entrepreneurial ability. In terms of employment and career selection, girls prefer stable and low-risk jobs, so they will not deliberately improve their entrepreneurial ability.

There are significant differences in the innovative spirit of college students in grades. The innovation spirit of students in four grades from high to low is senior > junior > sophomore > freshman; in other words, the higher grade is better than, the lower grade. There may be two reasons for this phenomenon. First, there is a specific relationship between innovative spirit and students’ intelligence and knowledge experience. Students with a high level of intelligence and more knowledge and experience tend to have a high spirit of innovation, dare to break the routine when faced with problems, and solve problems creatively. Therefore, compared with the students in the lower grades, after 4 years of study and knowledge accumulation, the upper grades have an enhanced ability to view problems comprehensively. Their innovative spirit is often higher. Second, this may be related to the level of entrepreneurship education students receive. Colleges and universities usually start to offer theoretical courses related to innovation and entrepreneurship in the first semester of the first year and gradually open related lectures and activities in the second year of freshman. In the junior and senior years, entrepreneurship and innovation education forms in schools are richer, including entrepreneurship projects and entrepreneurship plan competitions. Therefore, first-year students have less contact with entrepreneurship and innovation education in a single form. In contrast, junior and senior students have received innovation and entrepreneurship education for a long time and in various shapes and have been influenced more and deeply. Therefore, the innovative spirit of senior students is higher than that of junior students.

There are significant differences in the entrepreneurial ability of college students in grades. Specifically, the entrepreneurial capacity of sophomores, juniors, and seniors is higher than that of first-year students. Besides, the average entrepreneurial ability of sophomores, juniors, and seniors is gradually rising, but there is no significant difference. This may be related to the type of entrepreneurship and innovation education students are exposed to. First-year students are generally exposed to theoretical courses and have fewer opportunities for relevant practical activities. Students begin to participate in relevant projects and practical activities from sophomore year. Practical exercises have a significant impact on students’ development. Through practical activities, they constantly train themselves and improve their practical ability. Therefore, the entrepreneurial capacity of senior students is higher than that of junior students.

There are significant differences in the innovation spirit of college students with or without close relatives’ entrepreneurship. This is manifested in the innovative spirit of students with entrepreneurial behavior of close relatives, which is higher than that of students without entrepreneurial behavior of close relatives. This phenomenon may be because parents or family members are the most immediate contacts in students’ daily lives, and family education is also an indispensable part of students’ growth. Generally speaking, families with close relatives who start businesses are relatively open-minded. Parents will also support students’ innovation and entrepreneurship, imperceptibly promoting students’ innovative consciousness and stimulating their innovative spirit.

There are significant differences in the innovation ability of college students with or without close relatives’ entrepreneurship. The entrepreneurial power of students with close relatives’ entrepreneurial experience is higher than that of students without close relatives’ entrepreneurial experience. This may be because students with close relatives who start their businesses can visualize the concept of entrepreneurship through observational learning, increasing students’ entrepreneurial confidence to a certain extent and improving students’ entrepreneurial ability in related practices.

### Practical Activities Make the Most Outstanding Contribution to the Innovative Spirit and Entrepreneurial Ability

Through investigation and research, it is found that the theoretical courses, practical activities, and professional courses integrated into entrepreneurship and innovation education can positively affect the innovation spirit and entrepreneurial ability. However, there are differences in the degree of influence. Among them, practical activities significantly impact innovation spirit and entrepreneurial ability.

There are two reasons for the most significant contribution of practical activities to the development of students. First, practical exercises are novel and easily attract students’ attention and stimulate interest. Practical activities are usually carried out in entrepreneurial projects, entrepreneurial training, or entrepreneurial competitions. Students consciously constitute teams, voluntarily participate, and independently complete innovative research projects or practical activities through cooperative exploration under the guidance of teachers. Compared with theoretical courses and professional courses of mass entrepreneurship and innovation education, practical exercises are more vivid, specific, and attractive. It is easy to attract students’ attention, stimulate students’ curiosity, promote students’ active participation, and promote students’ development. Second, practical activities of entrepreneurship education implement the people-oriented concept of students. Students are the main body of learning, and teachers are only promoters and guides. In participating in practical activities, students need to rely on their strength to find problems, discuss, exchange ideas and solve problems in cooperation with team members. When students encounter complex issues, teachers provide the necessary support for students’ development to encourage students to explore independently. In this way, students have the opportunity to verify their theoretical knowledge in practice and constantly update their knowledge structure, which is conducive to stimulating students’ innovative consciousness and improving their comprehensive practical ability. As the carrier of systematically imparting the basic knowledge of innovation and entrepreneurship, the theoretical curriculum is dominated by teachers, which is not conducive to students’ initiative.

In contrast, although the theoretical curriculum of innovation and entrepreneurship education can also promote the development of students, its influence is poor than that of practical activities. The reasons may be as follows. First, this form of entrepreneurship and innovation education generally focuses on imparting the knowledge structure of the system and adopts the teaching method, which hinders students’ initiative. Second, the lag of educational effectiveness is particularly evident in theoretical teaching. An old Chinese saying goes, “It takes 10 years to grow trees but a 100 years to rear people.” This reflects the long-term continuity of educational work and the lag of academic results. Students will not change immediately after receiving an education but need a specific time to understand, digest, absorb, apply, and finally internalize it into their knowledge reserve or gradually reflect it in their behavior. Theoretical teaching is different from skill training or practical activities. It has a subtle influence on students by imparting knowledge, increasing knowledge, and broadening their horizons. This effect is far-reaching and long-lasting but not significant in the short term.

However, colleges and universities should not neglect theoretical curriculum education because academic curriculum education plays a fundamental role in promoting students’ long-term development. Scientific literacy is the basis for developing an innovative spirit and entrepreneurial ability. The academic curriculum of innovation and entrepreneurship education is a crucial way to improve students’ scientific literacy, develop students’ intelligence, stimulate students’ innovative spirit, and improve students’ entrepreneurial ability. Without extensive theoretical knowledge of science and culture, students cannot obtain a comprehensive understanding of a particular field and cannot break through the limitations of the original cognition and make innovations. Therefore, theoretical courses are the foundation of students’ long-term development. The stronger the basis of students, the longer-term development. Consequently, it is essential to combine academic courses and practical activities to promote students’ innovative spirit and entrepreneurial ability. Only in this way can students creatively solve problems and improve functional capacity based on multitudes of scientific and cultural knowledge.

## Conclusion

At this stage, China is experiencing leapfrog development. Opportunities and challenges coexist, and the demand for innovative talents is far greater than that of other countries. Colleges and universities naturally undertake this crucial historical mission as a strong base for cultivating and transporting talents for society. Innovation and entrepreneurship education in colleges and universities is a necessary measure for China’s higher education to comply with the major strategy of developing an innovative economy and building an innovative country. As a frontier group accepting new ideas, concepts, and technologies, college students are the masters of the future society, the builders and promoters of national development, and the main participants and beneficiaries of innovation and entrepreneurship education in colleges and universities. Through entrepreneurship and innovation education, colleges and universities stimulate students’ innovative thinking, shape their innovative personality, encourage students to innovate and start businesses, help students achieve self-development, improve students’ comprehensive quality, and provide a solid intellectual support for constructing an innovative country.

This report aims to explore the moderating effect of comic education and LOC in innovation and entrepreneurship education in colleges and universities. Firstly, the significance of comics for innovation and entrepreneurship education is briefly discussed, and the existing innovation and entrepreneurship education mode in colleges and universities in China is introduced. Then, a simple comparative analysis is conducted on the internal and external LOC. On this basis, a research survey is performed on the innovation and entrepreneurship ability of college students. The research results indicate that college students’ innovation ability and entrepreneurship ability in China are medium-level, and the innovation ability is stronger than the entrepreneurship ability. This also shows that colleges in China pay too much attention to the theoretical education of innovation and entrepreneurship and ignore the practice. Besides, external factors considerably impact students’ innovative and entrepreneurial ability. In addition, students’ personal characteristics and the teaching quality of innovation and entrepreneurship in schools also significantly affect students’ innovation and entrepreneurship ability. This study aims to help colleges and universities to improve the entrepreneurship and innovation education system and the effectiveness of entrepreneurship and innovation education. Students are the experiencers and beneficiaries of entrepreneurship and innovation education in colleges and universities. Their feelings and development can more directly reflect the practical situation of entrepreneurship and innovation education to allow the school to understand the deficiencies in entrepreneurship and innovation education in time. Accordingly, schools can make targeted rectification through the feedback from students and provide operational guidance suggestions for theoretical courses, practical activities, and professional courses integrated into entrepreneurship and innovation education.

Future research will focus on two aspects. First, time-series data will be used for analysis when investigating the impact of entrepreneurship and innovation education on students’ development to see the growth and changes of students more clearly. Secondly, the sample size and coverage will be expanded based on the existing research to make the conclusions more representative. Finally, some questions will be added to the self-made scale to summarize the actual situation of schools and teachers to make the information more perfect and diverse.

## Data Availability Statement

The raw data supporting the conclusions of this article will be made available by the authors, without undue reservation.

## Ethics Statement

The studies involving human participants were reviewed and approved by Nanjing Normal University Ethics Committee. The patients/participants provided their written informed consent to participate in this study. Written informed consent was obtained from the individual(s) for the publication of any potentially identifiable images or data included in this article.

## Author Contributions

All authors listed have made a substantial, direct, and intellectual contribution to the work, and approved it for publication.

## Conflict of Interest

The authors declare that the research was conducted in the absence of any commercial or financial relationships that could be construed as a potential conflict of interest.

## Publisher’s Note

All claims expressed in this article are solely those of the authors and do not necessarily represent those of their affiliated organizations, or those of the publisher, the editors and the reviewers. Any product that may be evaluated in this article, or claim that may be made by its manufacturer, is not guaranteed or endorsed by the publisher.
